# Empowering access: the U.S.’ first over-the-counter birth control pill revolutionizes contraception

**DOI:** 10.1097/MS9.0000000000001986

**Published:** 2024-03-21

**Authors:** Fatima Bint Sajid, Zaib Un Nisa Mughal, Nabiha Syed, Abdul Malik, Abdullah Mussarat, Burhanuddin Sohail Rangwala, Syeda Mahrukh Fatima Zaidi, Hussain Sohail Rangwala, Mirha Ali, Asma Ahmed Farah

**Affiliations:** a Department of Medicine, Jinnah Sindh Medical University; b Department of Medicine, Dow University of Health Sciences, Karachi, Sindh, Pakistan; c Department of Medicine, East Africa University, Bosaso, Somalia

## Introduction

Birth control pills are considered one of the most remarkable interventions for preventing unwanted pregnancies since their introduction into the market. Oral contraceptive pills are either marketed as combined oral contraceptive pills (COCPs) containing estrogen and progesterone or progesterone-only pills (POPs). Around 25% of women between the ages of 15 and 44 are reported to use contraceptive pills as their method of choice^1^. Despite their popularity, access to birth control pills comes with barriers. A study was conducted using a web panel to assess the factors that made accessibility to contraceptive pills harder. Some hurdles included getting an appointment with the clinician, lack of insurance, and access to a pharmacy. Overall, one-third of the women who took the survey reported barriers to accessing contraceptive pills^2,3^. The FDA, in its news release, mentioned that over half of the 6.1 million pregnancies in the United States (U.S.) are unintended each year^4^.

The article aims to discuss the significance of the FDA’s approval of the first over-the-counter (OTC) birth control pill in the U.S., explore the potential benefits and implications of this decision for reproductive health and family planning efforts, and provide insights into the features, efficacy, and safety of the newly approved contraceptive pill, norgestrel.

### Norgestrel

Norgestrel, a 0.075-mg tablet under the name Opill, has been approved by the FDA as an OTC contraceptive pill nationwide^5^.

Norgestrel differs from the commonly used COCPs as it contains only progestin, while regular COCPs contain estrogen and progesterone. Norgestrel works by preventing the ovulation process in the ovaries. Progesterone undergoes a negative feedback mechanism inhibiting the hypothalamus from secreting pulsatile gonadotropin-releasing hormone (GnRH). This, in turn, stops the production of follicle-stimulating hormone (FSH) and luteinizing hormone (LH) from the pituitary gland, thus preventing the maturation of follicles in the ovaries. Norgestrel also produces thick mucus in the cervix, making it hard for the sperm to transport to the fallopian tubes. While most POPs have various uses (e.g. PCOS – polycystic ovary syndrome), norgestrel has only been approved by the FDA to prevent pregnancy, that is, as a contraceptive^1,4^.

Unlike the conventional COCPs, its ease of availability and the potential to be a safe contraception for all age groups makes it a widely acceptable mode of contraception, especially amongst teenagers. Norgestrel is a critical advancement for better women’s reproductive health care, but experts fear its approval might face legal challenges. The cost of norgestrel hasn’t been announced yet, and its coverage by insurance also remains unclear. Still, its safety and effectiveness have harnessed the pill’s excellent media coverage before its presence as an OTC medication^5^.

Contraceptive pills to prevent unwanted pregnancies have provided women with significant autonomy regarding their reproductive health, and making one such drug available over the counter to the mass population without any barrier or discrimination will further help women of all age groups to make better decisions regarding their reproductive lives. It has more efficacy compared to the present OTC contraceptives available and gives similar results in breastfeeding women, making it a suitable contraceptive for them.

Eight U.S. clinical trials were conducted, and around 98 of 100 sexually active women who took norgestrel for a year reported not becoming pregnant^6^. Norgestrel is a very safe pill and shows no association with cardiovascular diseases, including venous thrombotic events (VTE), hypertension, cancer, or congenital disabilities. It has numerous advantages as an OTC medication and has fewer side effects making it a widely acceptable choice for women^6^.

## Adverse events

While COCPs are notoriously known for causing bleeding in women and increased systemic symptoms, POPs like norgestrel have far fewer symptoms. They are generally considered safe for use by all age groups. Systemic symptoms of COCPs include nausea, abdominal cramps, headaches, breast tenderness, and increased vaginal discharge. COCPs can also cause hypertension in 4–5% of healthy females. The risk of VTE increases during the first year of COCP intake^1,7,8^.

A meta-analysis determined the association between COCPs usage and myocardial infarction. COC users were concluded to have higher chances of getting an MI compared to non-COC users: relative risk (RR) 1.6 (95% CI 1.3–1.9)^9^.

POPs have fewer systemic symptoms compared to COCPs. POPs like norgestrel usually alter the menstrual cycle, leading to irregular periods, spotting between periods, or stopping periods completely. Norgestrel intake can occasionally lead to nausea, dizziness, headaches, increased appetite, abdominal pain, or cramps^10^.

Contraceptive pills, both COCPs and POPs, do not protect against HIV or sexually transmitted diseases (STDs)^1,10^.

## Direction to use and preference

Norgestrel is a very easy-to-use medication and comes with clear directions. One pill should be taken daily at the same time to prevent any unwanted pregnancy. The pill should be taken even if one bleeds, and the next packet of pills should be started immediately after the last one without any gap.

If a tablet is missed for one or more days, it should be taken immediately as one remembers their missing dose. The rest of the pills should be taken in the usual manner. If intercourse occurs during this period, another contraceptive barrier should be used (e.g. a condom) for the next 2 days, as the pill becomes effective after taking it for 2 days.

Another contraceptive barrier should be used for the coming 2 days whenever:

- One has only started taking the new pill pack.
- One remembers her missing dose.
- One vomits or has diarrhea within 4 h of taking the pill^10^.

Women may prefer oral contraceptives over other methods like intrauterine devices (IUDs) because they are easier to take than simply swallowing a pill as opposed to undergoing a medical procedure for insertion by medical professionals or health care providers, and it’s much easier than being injected. Additionally, oral contraceptives don’t carry the risk of pelvic infections that can occur with prolonged use of IUDs. This ease of administration and reduced risk make oral contraceptives a more appealing option for many women^11^.

## Limitations

Norgestrel, a POP, has fewer contraindications than traditional COCPs. These include the history of breast cancer, pregnancy, use of another birth control pill, or norgestrel as an emergency contraceptive pill^10^. Norgestrel can be taken by breastfeeding mothers, unlike the COCPs. It is a safe drug and does not affect the child’s health^10^.

## Conclusion

In conclusion, the FDA’s approval of the first OTC birth control pill in the U.S. represents a momentous achievement in advancing reproductive healthcare accessibility. This decision can empower individuals with greater control over their reproductive choices by making birth control more readily available without requiring a prescription. The availability of an OTC birth control pill may increase convenience and affordability and improve public health outcomes, contributing to more effective family planning efforts. This landmark decision is expected to positively impact the well-being and autonomy of individuals seeking contraceptive options.

## Ethical approval

Ethics approval was not required for this editorial.

## Consent

Informed consent was not required for this editorial.

## Sources of funding

The authors received no extramural funding for the study.

## Author contribution

F.B.S. and B.S.R.: conceptualization; N.S., Z.U.N.M., F.B.S., A.Maalik, A.Mussarat, H.S.R., M.A., and A.A.F.: literature and drafting; B.S.R.: editing and supervision. All authors have read and agreed to the final version of the manuscript.

## Conflicts of interest disclosure

The authors declare no potential conflicts of interest concerning the research, authorship, and/or publication of this article.

## Research registration unique identifying number (UIN)

Not applicable.

## Guarantor

Not applicable.

## Data availability statement

Not applicable.

## Provenance and peer review

Not applicable.
